# Actionable, long-term stable and semantic web compatible identifiers for access to biological collection objects

**DOI:** 10.1093/database/bax003

**Published:** 2017-02-26

**Authors:** Anton Güntsch, Roger Hyam, Gregor Hagedorn, Simon Chagnoux, Dominik Röpert, Ana Casino, Gabi Droege, Falko Glöckler, Karsten Gödderz, Quentin Groom, Jana Hoffmann, Ayco Holleman, Matúš Kempa, Hanna Koivula, Karol Marhold, Nicky Nicolson, Vincent S. Smith, Dagmar Triebel

**Affiliations:** 1 Botanic Garden and Botanical Museum Berlin, Freie Universität Berlin, Berlin, Germany; 2 Royal Botanic Garden Edinburgh, Edinburgh, UK; 3 Museum für Naturkunde Berlin, Leibniz-Institute for Evolution and Biodiversity, Berlin, Germany; 4 Museum National d'Histoire Naturelle Paris, Paris, France; 5 CETAF-Consortium of European Taxonomic Facilities, Brussels, Belgium; 6 Botanic Garden Meise, Meise, Belgium; 7 Naturalis Biodiversity Center, Leiden, The Netherlands; 8 Institute of Botany, Plant Science and Biodiversity Center, Slovak Academy of Sciences, Bratislava, Slovakia; 9 Finnish Museum of Natural History, University of Helsinki, Helsinki, Finland; 10 Department of Botany, Faculty of Science, Charles University, Praha, Czech Republic; 11 Biodiversity Informatics & Spatial Analysis, Royal Botanic Gardens, Kew, London, UK; 12 Department of Life Sciences, The Natural History Museum, UK; 13 SNSB IT Center, Staatliche Naturwissenschaftliche Sammlungen Bayerns, München, Germany

## Abstract

With biodiversity research activities being increasingly shifted to the web, the need for a system of persistent and stable identifiers for physical collection objects becomes increasingly pressing. The Consortium of European Taxonomic Facilities agreed on a common system of HTTP-URI-based stable identifiers which is now rolled out to its member organizations. The system follows Linked Open Data principles and implements redirection mechanisms to human-readable and machine-readable representations of specimens facilitating seamless integration into the growing semantic web. The implementation of stable identifiers across collection organizations is supported with open source provider software scripts, best practices documentations and recommendations for RDF metadata elements facilitating harmonized access to collection information in web portals.

**Database URL**: http://cetaf.org/cetaf-stable-identifiers

## Introduction

Natural history institutions worldwide are estimated to hold >2.5 billion physical collection objects (1). Together, they form an indispensable and essential resource for documenting and understanding of the occurrence of organisms in space and time (2). Besides the core data on taxon identification, geographic location, collector and time of the collection event, biological specimens often include important ecological and morphological details as well as annotations, which further increase their value for numerous biodiversity research questions. Finally, the act of naming taxa includes the assignment of one or several ‘type specimens’, which, from that point on, serve as an unambiguous reference for the taxon name.

Over the last decades, a global biodiversity data infrastructure has been erected providing open and instant access to digitized primary information provided by hundreds of natural history collections. By August 2016, biodiversity information networks such as GBIF (http://www.gbif.org), BioCASe [(3), http://www.biocase.org] or SpeciesLink [(4), http://splink.cria.org.br/], provide access to approximately 650 million occurrence records, 125 million of which belong to individual physical specimens held by museum or university collections. Searching this information space is mediated by a wide range of portals serving specific thematic or geographic user requirements based on a set of globally accepted data access protocols such as the BioCASE-Protocol (5), Distributed Generic Information Retrieval (DiGIR, http://digir.sourceforge.net), and the TDWG Access Protocol for Information Retrieval [TAPIR (6)] together with the community data standards Darwin Core (7) and ABCD [Access to Biological Collection Data (8)].

The stable and precise referencing of specimens used in scientific studies require the use of an agreed consistent system of globally unique identifiers (GUIDs). Particularly, the increased use of collection information in data-driven studies using advanced workflow systems such as Taverna (9) or Kepler (10) demand robust identifier specifications and implementations as a basis for data retrieval, integration and reproducibility of data experiments.

The natural history community acknowledges the need for an identifier system that meets the needs of handling specimen information in biodiversity research environments and has debated the use of different technologies such as LSIDs, DOIs, GBIF ‘Triple IDs’ and HTTP URIs (11). Unfortunately, the discussion has never been concluded so that only few collections have established a stable identifier system and existing systems differ considerably as to the choice of technology and implementation details (12).

In 2012, the Royal Botanic Garden Edinburgh implemented an effective working system of stable identifiers for herbarium specimens based on HTTP URIs and Linked Open Data principles (13). In addition to their function as stable anchors for referencing specimens, requests are redirected to different human readable and machine processable representations of the physical objects and their corresponding metadata. In 2013, the Consortium of European Taxonomic Facilities (CETAF) agreed to use this approach for the implementation of a joint HTTP URI based identifier system and to start pilot implementations and specification activities. The implementation received support from the pro-iBiosphere (http://www.pro-ibiosphere.eu) initiative aiming to prepare the ground for a global Open Biodiversity Knowledge Management System.

At present, 13 CETAF member institutions have implemented stable URIs. Establishing the system at 80% of CETAF institutions is part of the CETAF Strategy and Strategic Development Plan for this decade 2015–2025 (14). The new CETAF stable identifiers are increasingly used for referencing specimens in taxonomic publications, data portals, and web service interfaces. In addition, several pilot projects are underway to demonstrate the integration into Linked Open Data based applications.

## Methods

### Syntax of HTTP URIs for collection objects

The basic generic syntax of Uniform Resource Identifiers (URIs) for identifying resources is formally described in the current Standard RFC 3986 (15). This syntax is very open in the sense that basically only the fundamental building blocks (scheme, authority, path, query and fragment) and their arrangement are specified. For particular applications of URIs the generic identifier can be further restricted to more specific formal grammars for example to support access protocol agreements.

**Figure 1.**
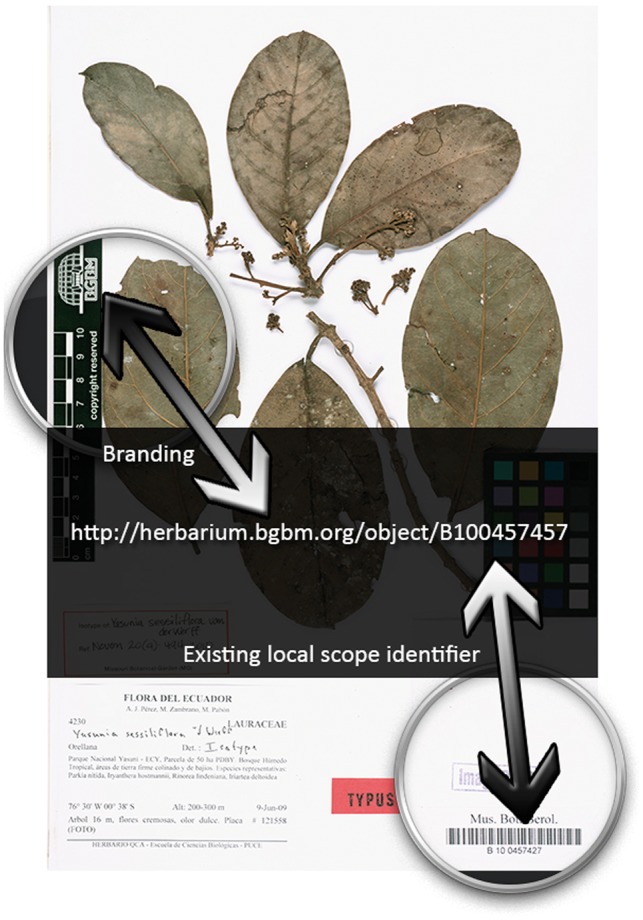
Example specimen. Example physical herbarium object and its stable HTTP URI identifier.

CETAF organizations agreed on using HTTP URIs for the identification of physical collection objects as well as associated information resources (e.g. multimedia, RDF, web pages), because they are the dominant mechanism to reference information in the World Wide Web. It was further agreed that more restrictive syntax specifications prescribing precisely the structure of identifiers for natural history collection objects across institutions would hinder the implementation process and should be avoided. Instead, participating institutions collaboratively developed a best practices document, including basic recommendations and background information on HTTP URIs, non-binding recommendations on URI patterns applied to the Semantic Web and Linked Open Data, examples which can be used as a blueprint for own implementations, as well as a list of concrete patterns used by early implementers in the natural history domain (16). The flexibility to choose their own syntax together with the documentation of recommended patterns was the main reason for the rapidly growing number of working implementations in CETAF institutions. The typical URI pattern is composed of the institutions' web domain, a meaningful subdomain, a class (within which similar objects may be found), and an existing local object identifier (such as an object barcode) (Figure 1).

The web domains owned and controlled by the respective institutions ensure the global uniqueness of the identifiers and thus, the local object identifiers only need to be unique within each institution's scope which all Institutions are capable to achieve. As the full string is treated as the identifier rather than just the local ID, HTTP URIs can be considered as highly persistent as long as global domain name registrars guarantee the uniqueness of the domain names throughout the World Wide Web.

### Basic identification and redirection mechanisms

**Figure 2.**
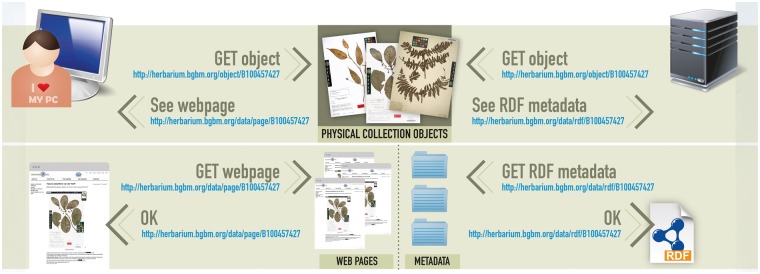
Basic redirection mechanisms. Human users are redirected to a human-readable web-representation of the specimens. Software systems are re-directed to a machine-readable metadata record.

With the availability of CETAF stable identifiers, both physical and digital specimens and their associated information resources can be uniquely and persistently referenced. The distinction between the specimen itself and the information resources containing metadata describing this object (e.g. taxon name, collector and provenance) is occasionally omitted in oversimplified information models and data portals. However, this is a prerequisite for a consistent system, which enables different users to talk about the same specimen or discuss (and perhaps reject) claims made in different information resources about this specimen. Assertions that refer to objects by means of identifiers can be integrated and provide a basis for the inference capabilities of the Semantic Web. The identifiers’ full potential unfolds with the implementation of redirection mechanisms. Users trying to access a specimen using a web-browser and the specimen identifier will be redirected to a human-readable representation of the objects, typically an HTML-webpage. On the other hand software-systems requiring machine-readable representations of the object will be redirected to a (preferably) RDF-encoded metadata record (Figure 2).

The redirection mechanisms have to be setup locally by participating organizations. For ease of implementation working example PHP scripts are provided on a SourceForge site (https://sourceforge.net/projects/stablecollectionidentifiers), which can easily be modified to meet the local technical requirements. The software includes a function for transforming specimen metadata into a simple set of RDF elements mainly consisting of concepts defined by the Darwin Core and Dublin Core (17) standards. However, participating institutions are free to use their internally developed software and different sets of RDF elements.

### Quality of service

An essential aspect of the new identifier system is that participating organizations keep their identifiers stable and ensure that redirection mechanisms are working reliably. The fact that stable, web-enabled identifiers are just as important as the identifiers locally used on physical objects (e.g. museum numbers or barcodes) has to be recognized in the development of curation strategies for natural history collections. The awareness of the need for appropriate management procedures is already growing with the increasing use of CETAF stable identifiers in taxonomic workflows.

## Results

As of June 2016, the following 13 organizations have implemented CETAF stable identifiers for their collections:

- Botanic Garden and Botanical Museum Berlin
- Finnish Museum of Natural History, Helsinki
- Institute of Botany, Slovak Academy of Sciences, Bratislava
- Museum für Naturkunde Berlin
- Muséum national d'histoire naturelle, Paris
- Naturalis Biodiversity Center, Leiden
- The Natural History Museum, London
- Natural History Museum, University of Oslo
- Royal Botanic Garden Edinburgh
- Royal Botanic Gardens Kew, London
- Staatliche Naturwissenschaftliche Sammlungen Bayerns
- Staatliches Museum für Naturkunde Stuttgart
- Zoologisches Forschungsmuseum Alexander Koenig, Bonn

**Figure 3.**
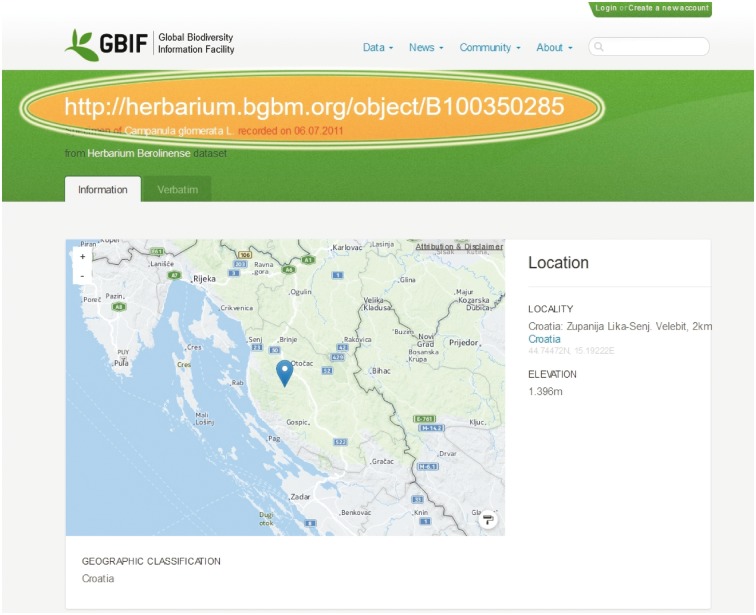
CETAF stable HTTP URIs in the GBIF data portal. The Global Biodiversity Information Facility (GBIF) publishes CETAF stable HTTP URIs via their data portal.

The new identifiers are increasingly accepted by the scientific community and used in scientific publications referencing specimens as well as in institutional web-based query systems and large scale information systems such as the data portal of the Global Biodiversity Information Facility GBIF (Figure 3, http://www.gbif.org) and the Global Genome Biodiversity Network [GGBN, (18)].

**Figure 4.**
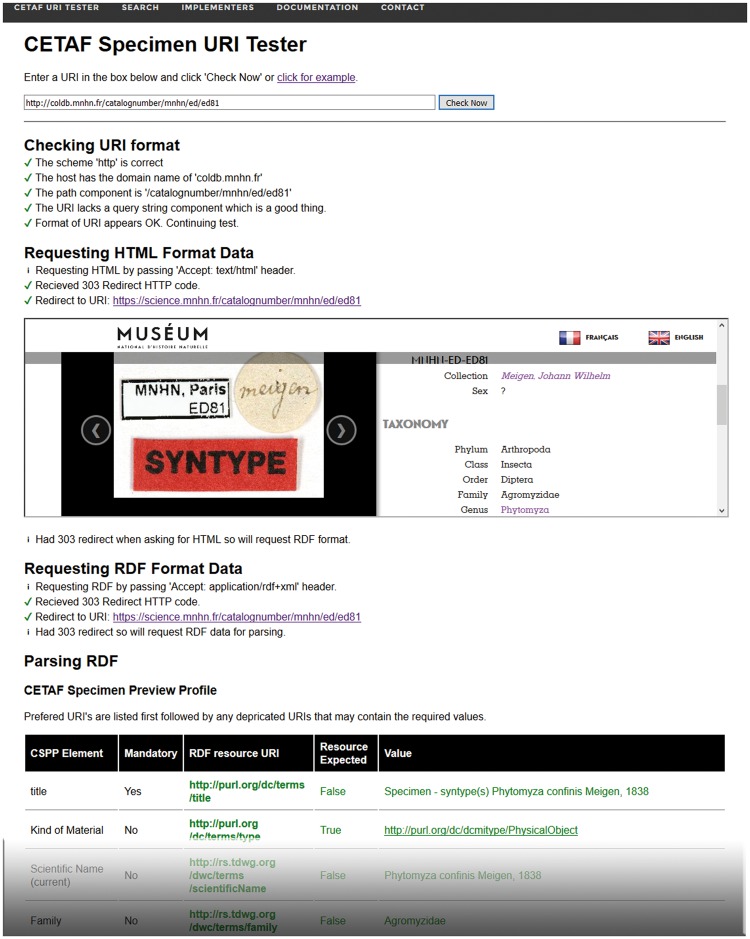
The CETAF Specimen URI Tester provides for any given Specimen URI an overview of the redirection process as well as a preview of machine-readable and human-readable data associated with the URI.

To support quality control measures, a capable and easy to use ‘CETAF Specimen URI Tester’ (http://herbal.rbge.info) has been implemented (Figure 4). The web-based system provides for any given URI the following tests:

- Syntax check of the URI format.
- Testing of redirection to human-readable content.
- Preview of human-readable content.
- Testing of redirection to machine-readable content.
- Testing of availability of specific data elements and preview.
- Preview of complete RDF-Graph.

The system is highly useful for testing both the technical implementation and semantic aspects of mapping local collection data to RDF standards used by the international biodiversity informatics infrastructures.

Working implementations of the stable identifier systems are registered in the URI tester as well as on the CETAF website (http://cetaf.org/cetaf-stable-identifiers). Each registration contains an example identifier as well as a link to a local dynamic catalogue if implemented.

Although there is no binding set of mandatory RDF metadata elements to be provided by implementing organisations, it was agreed that client applications can benefit from a defined set of recommended metadata elements which can be used to generate meaningful previews of specimens and their metadata. The ‘CETAF Specimen Preview Profile’ (CSPP) constitutes such an element set composed of 13 agreed and easy-to-provide Darwin Core and Dublin Core concepts considered useful for preview functions, such as decorated links and search results, in data portals. The CSPP-elements are title, kindOfMaterial, scientificNameCurrent, family, scientificNameOriginal, collectorNumber, collectorName, webscaledImageLink, latitude, longitude, isoCountry, collectionDate and sourceLink (19).

**Figure 5.**
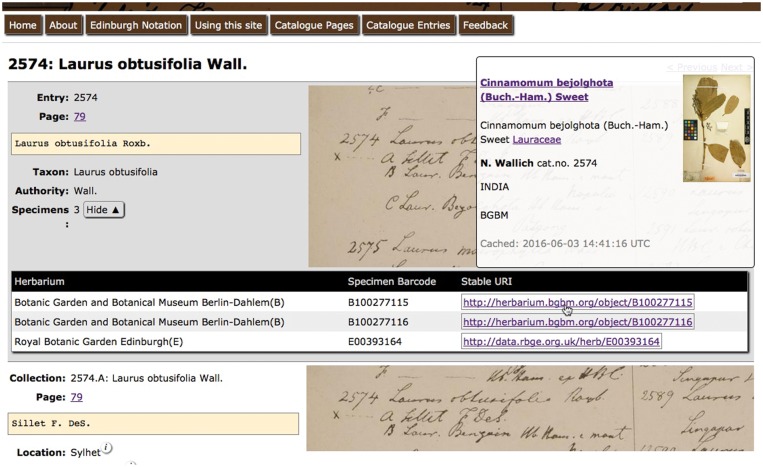
The Wallich Catalogue. Screenshot of Wallich Catalogue hosted by Royal Botanic Garden Edinburgh showing popup for stable URI containing information hosted at Botanic Garden and Botanical Museum Berlin-Dahlem.

In addition, the capabilities of Linked Open Data to facilitate cross-institutional thematic information systems are demonstrated on the example of the ‘Wallich Catalogue Online’ system developed by the Royal Botanic Garden Edinburgh. The Catalogue provides an interactive tool helping researchers understand the Wallich Catalogue and interpret the herbarium specimens Nathaniel Wallich distributed on behalf of the British East India Company between 1829 and 1847 (Figure 5, http://wallich.rbge.info). The informatics challenge this project faced was to allow users to browse specimen information from multiple herbaria without building a new database that duplicated specimen information. The system's focus is on curating information about the catalogue whilst leaving individual herbaria to curate authorative information on the specimens they hold. The solution adopted was to store only the HTTP URIs for the remote specimens in the database and pull specimen data in when the user required it. For efficiency, the specimen data is cached briefly but not stored in the database. Data supplied in a CSPP response are used to display core specimen information, a thumbnail image and a link to the original source. An example of the use of this functionality is quickly spotting specimens that don’t look like they are true duplicate specimens simply by hovering the mouse over the Wallich Catalogue page entry.

## Discussion and outlook

In this paper, we have provided an overview of the status of the initiative of the Consortium of European Taxonomic Facilities CETAF to implement an agreed system of stable HTTP-URI-based identifiers for specimens. The system has been successfully implemented in 13 member institutions. Best practices documentations and reusable open source software components support institutions which now want to join. The new identifiers are already used in publications and data portals. A pilot system has been put up demonstrating the integration of distributed collection information using the Linked Open Data capabilities of HTTP URIs.

Apart from implementing CETAF stable identifiers in further organizations, the next steps will focus on improving the interoperability of linked open collection data. This involves (i) the development of machine-readable catalogues of specimens, which can be used as an access point for harvesting metadata, (ii) the development of aggregators facilitating fast indexing and searching across collections and (iii) linking out from collection data to external information resources describing, for example, scientific names, geographic entities and persons. The implementation of a central index will provide the ground for effective quality control measures which are difficult to realise in a purely distributed system. A first prototype of such an index has been integrated into the URI tester.

The extension to other physical and virtual object types should be considered. With this, the Natural History Collection domain will bridge the gap to other disciplines and open the door to new ways of accessibility, semantic enrichment and inference.

CETAF stable identifiers are likely to play an important role also in referencing to particular specimens in formal nomenclature acts (both in publications publishing these acts and in respective databases) that are performed in accordance with the International Code of Zoological Nomenclature (20) and the International Code of Nomenclature for algae, fungi and plants (21). Currently a web portal for registration of new and past lectotypifications is being developed and stable identifier link to the newly (or past) designated lectotypes of names are likely to be very useful permanent references to specimens. There are already examples of papers using CETAF stable identifiers in formal nomenclature acts (22, 23).

Stable identifiers will have numerous advantages for researchers, data managers, collection managers, policy-makers and institutions. For example, the delay between information gathering and creating actionable evidence for conservation is slow. Stable identifiers can underpin reproducible workflows for research, because they facilitate the automation of processes without losing the providence of data. Such automated workflows can be repeated as soon as new data become available, shrinking the time between observation and action, but also reducing uncertainty.

Another potential of stable web URIs dynamically generated from 2D, QR- or matrix-code accession numbers pinned or glued on the physical objects lies in their integration into collection workflows. A web query for identifiers, done by researchers and collection managers working in the collection magazines, e.g. using mobile apps, will return the direct link to web pages with accredited, object-related information—after this information is published by the object-owning institution.

Stable identifiers interlink objects and their metadata, but also help link different data together. They therefore facilitate data discovery and can be used to monitor the usage and impact of objects and collections. Furthermore, by linking specimens held at different institutions, stable identifiers will expedite the sharing of data, thus reducing curation costs and improving data quality. This will promote taxonomic precision by allowing specimens to be cited with greater ease leading to more specimen based monographic approaches (24).

Stable identifiers for specimens are an important step in improving the interoperability of biodiversity data. However, it imposes long-term responsibilities on the providing institutions, their researchers, curators, publishers and informaticians. They all need to understand the importance of these identifiers and that their stability has to be considered together with general curatorial practice. Specimens cannot simply be remounted, split, re-barcoded or deaccessioned without considering what impact this may have on the stable identifier. Institutional data policies and data management plans may be a useful tool to ensure that these issues are communicated and can be used to detail procedures that ensure stability. For example, given the sensitivity of institutional branding and the occasional merging of collections and exchanging of specimens, for some institutions using weakly branded identifiers might be preferable for the sake of future stability.

If collections are split or merged, then the responsibility to maintain the persistence of URI may falls to another organisation. To maintain continuity in the long term there are costs, the persistence of URIs cannot be seen as an adjunct to current practise, but a core responsibility of curators and institutions. To this end it is important that software for collections management, data analysis, aggregation and publication should support the maintenance and uniqueness of these identifiers. This is particularly important for smaller collections who, without access to IT support staff, need off-the-shelf solutions to support persistent URIs.

The responsibility imposed to the institutions includes maintaining their domain names for long-term. Moving an object from one collection to another may imply a valid change of the object's identifier. The former owner then needs to redirect to the other collection's new URI with HTTP code 301 (‘permanently moved’) or at least needs to refer to the changes in the metadata for the case where the new owner does not use HTTP URIs. In any case, the ‘old’ identifier needs to be preserved to meet the requirement of persistency.

Political changes (e.g. renaming of the institution or changes of country's top-level domains) might still influence the persistence of the URIs and there is no easy solution. Awareness of the problem will encourage institutions to select their identifier syntax thoroughly to avoid instabilities as far as possible. Using centralized systems like the services of PURL.org or DOI.org are fully compatible with the concept presented in this article, and institutions might choose one of those services in order to avoid changing components in the URIs. However, both PURLs and DOIs just move the URI persistence problem to a third party and break the notion of institutional responsibility that is vital to the social success of our approach.

## References

[1] Duckworth W.D. Genoways H.H. Rose C.L. (1993) Preserving Natural Science Collections: Chronicle of Our Environmental Heritage. National Institute for the Conservation of Cultural Property, Washington, DC.

[2] Chapman A.D. (2005) Principles of Data Quality, version 1.0. Report for the Global Biodiversity Information Facility, Copenhagen.

[3] Güntsch A. Mergen P. Berendsohn W.G. (2007) The BioCASE Project - a Biological Access Service for Europe. Ferrantia, 51, 103–108.

[4] Canhos V.P. Souza S.D. Giovanni R.D. (2004) Global Biodiversity Informatics: setting the scene for a "new world" of ecological forecasting. Biodiversity Informatics, 1, 1–13.

[5] Holetschek J. Kelbert P. Müller A. (2009) International Networking of Large Amounts of Primary Biodiversity Data. In: Fischer S, Maehle E, Reischuk R, editors. INFORMATIK 2009, Im Focus das Leben, Beiträge der 39. Jahrestagung der Gesellschaft für Informatik e.V. (GI), 28.9. - 2.10. in Lübeck. Lecture Notes in Informatics (LNI), **154**, 552–564.

[6] De Giovanni R. Döring M. Güntsch A. (2010). TDWG Access Protocol for Information Retrieval (TAPIR), Version 1.0. Biodiversity Information Standards (TDWG), http://www.tdwg.org/standards/449.

[7] Wieczorek J. Bloom D. Guralnick R. (2012) Darwin core: an evolving community-developed biodiversity data standard. PLoS One, 7, e29715. 22238640 10.1371/journal.pone.0029715 PMC3253084

[8] Holetschek J. Dröge G. Güntsch A. (2012) The ABCD of primary biodiversity data access. Plant Biosyst., 146, 771–779.

[9] Hull D. Wolstencroft K. Stevens R. (2006) Taverna: a tool for building and running workflows of services. Nucleic Acids Res., 34, 729–732. 10.1093/nar/gkl320 PMC1538887 16845108

[10] Altintas I. Berkley C. Jaeger E. (2004) Kepler: an extensible system for design and execution of scientific workflows. In: Scientific and Statistical Database Management, Proceedings. 16th International Conference on Scientiﬁc and Statistical Database Management, SSDBM ’04, San Diego, pp. 423–424.

[11] Richards K. (2009) TDWG GUID Applicability Statement, Version 2010-09. Biodiversity Information Standards (TDWG). http://www.tdwg.org/standards/150.

[12] Guralnick R.P. Cellinese N. Deck J. (2015) Community next steps for making globally unique identifiers work for biocollections data. ZooKeys, 494, 133–154. 10.3897/zookeys.494.9352 PMC4400380 25901117

[13] Hyam R.D. Drinkwater R.E. Harris D.J. (2012) Stable citations for herbarium specimens on the internet: an illustration from a taxonomic revision of Duboscia (Malvaceae). Phytotaxa, 73, 17–30.

[14] Consortium of European Taxonomic Facilities (2015) CETAF Strategy and Strategic Development Plan 2015–2025. http://cetaf.org/sites/default/files/final_strategy_and_strategic_development_plan.pdf.

[15] Berners-Lee T. Fielding R. Masinter L. (2005) Uniform Resource Identifier (URI): Generic Syntax. https://tools.ietf.org/html/rfc3986.

[16] Hagedorn G. Catapano T. Güntsch A. (2013) Best practices for stable URIs. http://wiki.pro-ibiosphere.eu/wiki/Best_practices_for_stable_URIs.

[17] Dublin Core Metadata Initiative: Dublin Core Metadata Element Set, Version 1.1. http://dublincore.org/documents/dces/.

[18] Droege G. Barker K. Astrin J. (2014) The global genome biodiversity network (GGBN) data portal. Nucleic Acids Res., 42, D607–D612. 24137012 10.1093/nar/gkt928 PMC3965106

[19] Anonymous (2015) CETAF Specimen Preview Profile. http://cetafidentifiers.biowikifarm.net/wiki/CSPP.

[20] Ride W.D.L. Cogger H.G. Dupuis C. (1999) International Code of Zoological Nomenclature. International Commission on Zoological Nomenclature, London.

[21] McNeill J. Barrie F.R. Buck W.R. (2012). International Code of Nomenclature for algae, fungi, and plants (Melbourne Code): adopted by the Eighteenth International Botanical Congress Melbourne, Australia, July. Koeltz Scientific Books, Königstein.

[22] German D.A. Tekın M. Španiel S. (2016) A brief taxonomic revision of Physoptychis (Alysseae, Brassicaceae). Phytotaxa, 258, 75–82.

[23] Marhold K. Kempa M. Al-Shehbaz I.A. (2015) Lectotypification of names of Himalayan Brassicaceae taxa currently placed in the genus Cardamine. PhytoKeys, 50, 9–23. 10.3897/phytokeys.50.5080 PMC4489080 26140016

[24] Pullan M.R. Watson M.F. Kennedy J.B. (2000) The Prometheus Taxonomic Model: a practical approach to representing multiple taxonomies. Taxon, 49, 55–75.

